# Gender and Its Role in the Resilience of Local Medical Systems of the Fulni-ô People in NE Brazil: Effects on Structure and Functionality

**DOI:** 10.1155/2019/8313790

**Published:** 2019-06-12

**Authors:** Wendy Torres-Avilez, André Luiz Borba do Nascimento, Flavia Rosa Santoro, Patricia Muniz de Medeiros, Ulysses Paulino Albuquerque

**Affiliations:** ^1^Departamento de Biologia, Programa de Pós-graduação em Etnobiologia e Conservação da Natureza, Universidade Federal Rural de Pernambuco, Recife, Brazil; ^2^Departamento de Botânica, Laboratório de Ecologia e Evolução de Sistemas Socioecológicos (LEA), Centro de Biociências, Universidade Federal de Pernambuco, Brazil; ^3^Centro de Ciências Agrarias, Universidade Federal de Alagoas, Maceió, Brazil

## Abstract

Ethnobotanical studies focused on understanding how local medical systems are functionally maintained suggest that utilitarian redundancy and knowledge transmission are factors that influence the resilience of the system. However, to date, there have not been any studies that analyze these factors in relation to the variables that influence the variation of knowledge. Given the above, this study aims to analyze the influence of gender in the resilience of the system, using utilitarian redundancy and knowledge transmission as factors. Information from 198 married couples (396 people) was collected from the indigenous community of Fulni-ô (NE Brazil). Knowledge between men and women was analyzed based on the total number of known plants, therapeutic targets, information units, utilitarian redundancy, models of transmission, and sharing for each gender. Fulni-ô men know a greater number of plants, therapeutic targets treated with plants, and information units than women. They also had greater utilitarian redundancy. However, regarding knowledge transmission, sharing among women was greater, transmission is related to gender, and there is no difference between the numbers of models of knowledge information. In the system of local medical knowledge, gender exerts an important role in the resilience of the system. This study shows that men have a greater contribution to the structure and function of the system; however, both genders contribute to the flow of information in the system, which makes both genders important in the feedback of information.

## 1. Introduction

Local medical systems (LMSs) involve the knowledge, actions, and specific beliefs of a local population related to health and disease protection, along with the social actors and local resources used for disease treatment [1, 2]. In this context, the role of social actors is important for the maintenance of this system, and the gender of these social actors has an influence on that role, especially regarding knowledge of medicinal plants [3–6]. Thus, an analysis of the LMSs based on the role of the social actors enables the essential aspects that contribute to the resilience of these systems to be identified. To this aim, the concept of resilience is relevant. This concept is derived from ecology and is understood as the capacity of a system to absorb a future disturbance by reorganizing its function, structure, and continuity so that it retains its identity [7, 8]. Initially, this concept was applied to ecological systems, but it is now being applied to social-ecological systems [9, 10], such as LMSs [11].

To better understand the resilience of LMSs, Ferreira Júnior et al. [12] suggest some important factors that must be analyzed: utilitarian redundancy and knowledge transmission. Utilitarian redundancy also comes from ecological systems [13] and was adapted by Albuquerque and Oliveira [14] to understand the resilience of LMSs from a functional perspective. According to the Utilitarian Redundancy Model, some species have the same utilitarian function (utilitarian redundancy) in a local community; i.e., they treat the same therapeutic targets (perception of illness by members of the community); therefore, if one species is locally extinct or is forgotten by the social actors, the redundant species can functionally ensure the treatment of therapeutic targets. This arrangement suggests that such redundancy helps in the maintenance of system functions, contributing to resilience.

Similarly, knowledge transmission is essential for maintaining existing treatments because if the knowledge is restricted to a few people and is not transmitted, when these people leave the system, a loss of knowledge would occur; consequently, the medicinal function would disappear [15]. The forms of knowledge transmission need to be considered since they influence the knowledge that each individual possesses. Knowledge transmission in the population is not random and is determined by the environment and social factors [16, 17]. Different types of information transmission between the model (person from whom information is copied) and the apprentice have been proposed, some of which are related to the degree of kinship between them: vertical transmission that is from parent to child; horizontal transmission is that between individuals of the same generation; from one to many when the models are leaders (local specialists) or teachers who teach the residents of the community; and from many to one when older individuals are the reference for learning [18, 19]. These types of transmission may be present in a single individual for a specific knowledge depending on their degree of interaction with other individuals in the community [20].

Based on these two proposed factors of resilience, several studies have been conducted to understand the resilience of LMSs [11, 14, 21, 22], but the manner in which the gender of the social actors may influence these factors has yet to be addressed, considering it is an important variable in the variation of the knowledge of medicinal plants and it can influence the different factors related to the resilience. Therefore, the aim of this study is to understand the relative contribution of each gender to the maintenance of the system.

Gender is a variable that involves cultural beliefs and distribution of resources at different levels, which generates patterns of behavior and organization of practices [23]. Several studies have indicated that, in several communities, from a perspective of the social role of gender, women in their role as housewives are responsible for the care of the family and are responsible for diagnosing disease and implementing the initial treatment [24, 25]. The responsibility that the role of women as caregivers in the family carries may influence the development of skills in health care as a result of greater stimulus to find resources for health care.

Considering the structural perspective, ethnobotanical studies mention that, in some communities, women possess a greater knowledge of medicinal plants to solve health problems compared with men [4, 26–28]. The knowledge of LMSs between genders in a functional perspective has not been analyzed taking into consideration the diversity of diseases treated with medicinal plants, utilitarian redundancy, and the transmission of knowledge. However, in the case of knowledge transmission, one study proposed the following hypothesis: learning occurs frequently among people of the same sex [29].

Thus, the lack of studies that analyze the variation of knowledge between genders that integrate a structural and functional perspective led us to investigate issues related to the factors that are involved in the resilience of LMSs. From the* structural *point of view, (1) women have greater knowledge of medicinal plants than men; and (2) women have a greater understanding of information units (IUs). From the* functional* point of view, (1) women have a greater knowledge of therapeutic targets associated with known plants compared to men; and (2) the therapeutic targets that women have knowledge of have greater utilitarian redundancy than targets known by men. Regarding knowledge* transmission* and its importance in resilience, (1) women obtain knowledge from more models of transmission compared to men; (2) the knowledge of women is transmitted more among women than among men; and (3) there is a greater sharing of knowledge among women than among men.

## 2. Materials and Methods

### 2.1. Study Area

The study was conducted in the indigenous community of Fulni-ô in the Águas Belas municipality, state of Pernambuco (Northeastern Brazil), located at 9°06′45′′ S and 37°07′15′′ W, 315 km from Recife, the capital of Pernambuco [30]. The indigenous territory comprises an area of approximately 11,500 ha [31]. The community is composed of 3,430 people registered in the health unit, according to information available to the head of the unit in 2014. The people are settled in two villages, one located 100 m from Águas Belas, called village Cede (aldea Cede), and the other located 4 km from the village, called Xixiaklá.

The community lies within the extent of Caatinga vegetation [32], which is associated with a semiarid climate (savannah). There are well-marked environmental variations, a dry and a rainy season and ranges from 15 to 20 m in the conserved areas and up to 10 m in areas that are exposed to anthropogenic conditions that have been affected by long dry periods [33]. In the Caatinga, the dominant botanical families are Fabaceae, Convolvulaceae, Euphorbiaceae, and Poaceae [34]. This vegetation type is important for the members of the indigenous community because native species of high cultural significance for them are established here and used medicinally for food and artisanal handiwork, as in the case of* Syagrus coronata* (Mart.) Becc.,* Myracrodruon urundeuva* (Engl.) Fr. All.,* Lippia* sp.,* Amburana cearensis* (Arr. Câm) A. C. Smith.,* Aspidosperma pyrifolium *Mart.,* Sideroxylon obtusifolium* (Roem. & Schult.) T.D. Penn.,* Maytenus rigida* Mart.,* Hyptis mutabilis* Briq., and* Ziziphus joazeiro* Mart. [35].

### 2.2. The Fulni-ô

The indigenous community Fulni-ô originated in the 18th century, after the establishment of indigenous groups in the vicinity of the Ipanema River, now located in the municipality of Águas Belas. The original groups were the Flowkassa, the Tapuya, the Brogadais, and the Fulni-ô, and with the passage of time, the groups organized into a clan society named Fulni-ô, which means “we are from the river” in the native Yaathê language, referring to the Ipanema River, which is located south of the village [36]. Currently, the indigenous community Fulni-ô is the only one in the northeast of bilingual Brazil; Portuguese and the native language “Yaathê” are the main languages [37]. The identity of the Fulni-ô is linked to the principle of “*Safenkia fotheke*”, which means union and respect, which reflects the notion of reciprocity between the social actors that form the collective of the community that encourages homogenization and justifies and explains the way to be a Fulni-ô within their social, political and cultural relationships [38]. Policy decisions are the responsibility of the* Pajé* and* Cacique* [38].

A peculiarity of the Fulni-ô indigenous people is the sacred ritual called “Ouricuri”, performed for three consecutive months, between September and December in the homonymous village. During the annual ritual “Ouricuri”, there are standard principles that should be followed, for which nonindigenous people cannot have access, as indigenous people are sealed in those cultural aspects [38]. In the Ouricuri village, there is a sacred tree, the “Juazeiro” (*Ziziphus joazeiro* Mart.), which only Fulni-ô men can approach; non-indigenous men and women are prohibited [36].

The livelihoods of the Fulni-ô are based on activities such as the production of handicrafts, agriculture, land, salaried jobs, retirement income, government support, and artistic shows (Toré and Kafona) [39]. Some of the activities performed are in the urban area of the neighboring city (Águas Belas). Producing handicrafts and shows are activities considered exclusive to Indians. Among the ready-made handicrafts are bags, hats, mats, and brooms that are constructed with “Ouricuri” leaves (*Syagrus coronata* Mart) [39]. Agriculture in the indigenous community does not generate much economic income; these activities are performed by the indigenous people, and production is for self-consumption [39].

The health system of the Fulni-ô consists of the integration of biomedical practices and local medicine. The presence of this articulation in the community is observed as the strengthening of the search for identity and ethnicity, with the local medical system as a base, since they are active in the construction of reality [40]. Indians have access to a health unit within the community and to health workers who serve all areas of the village. They also have access to commercial drugs from the health unit and pharmacies in the city of Águas Belas, along with a directory of natural resources and rituals related to culture to treat diseases [40].

The medical knowledge system of the Fulni-ô is distributed among different levels of community members, and there are also recognized specialists, such as the”rezadoras” or “rezadores”, midwives, and elders [35]. Since there is no variation in the knowledge between plant specialists and nonspecialists, knowledge is distributed among the members of the general community, because the use of local plant resources is a function of individual competences [41]. Diseases are first recognized in the home, where the first diagnosis and treatment are established and where the eldest use herbal remedies; if not effective, pharmaceutical remedies, usually suggested by relatives or neighbors, are tried [36]. The “rezadores” are called to heal the diseases related to the culture, such as the evil eye, evil spirits, and evil winds [36].

### 2.3. Data Collection

The sample was obtained from married couples in the Indian village of Fulni-ô, providing an equal number of men and women who represent the distribution of medicinal plant knowledge within each family. It is worth noting that the knowledge between married couples was analyzed, but not among singles, because their different social roles may result in possessing different knowledge than married men and women. Based on the total number of married couples in the village (563) registered with the local health unit, a representative sample was selected using a 95% confidence level. This representative sample was divided between regions of the village, based on the division made by the health unit for attending to the 14 health workers; thus, our sample encompasses the entirety of the village. From the sample, 229 married couples were selected (458 people), of whom 14% declined to participate in the study, giving a total of 198 married couples (396 people).

The information was obtained by means of semistructured interviews accompanied by an indigenous person to facilitate the relationship between researchers and interviewees and to assist in the translation of plant names that were often remembered only in the Yaathê language. The interviews were conducted according to the schedule during which nonindigenous people can circulate in the village, between 9:00 and 12:00 and between 14:00 and 17:00, which also respected the Ouricuri ritual and the sacred days in which Indians are summoned to be gathered in the village, often twice a week.

Information was obtained from semistructured interviews by questioning participants about well-known medicinal plants, their therapeutic targets, and the transmission model from which knowledge was obtained. To obtain more information about transmission models, different methodologies were proposed, to avoid favoring parental transmission models [20]. In this study to obtain more specific information with semistructured interviews, the transmission model was asked for each plant-therapeutic target pair, which required an effort on the part of the person interviewed to remember the moment of learning. Models of learning refer to persons from which knowledge of medical plants was obtained, such as “great-grandmother”, “great-grandfather”, “grandmother”, “grandfather”, “mother, “father”, “uncle”, “aunt”, “brother”, “sister”, and others.

The plants mentioned by participants were collected together with the local parataxonomists (Antonio Verissimo, Renildo Paulo de Macedo, and Jemerson Caetano de Sá). The specimens were processed, identified, and stored in the Dardano of Andrade Lima Herbarium of the IPA (Instituto Agronômico de Pernambuco). The collection of botanical material was authorized by the SISBIO (Sistema de Autorização e Informação de Biodiversidade). Species sold for their consumption at different scales were not collected since their taxonomic identities are well known and herbaria do not easily accept them; therefore, the names of these species in this study were attributed based on their known taxonomic identity.

The local names of the plants reported by informants were used to data analysis. Synonyms of plant names were excluded with the help of the parataxonomist to avoid duplications. A species analysis was not conducted due to the small sample number collected resulting from a prolonged drought over the two years of the study. The indigenous community did not permit publication of the specific use of each plant; thus, out of respect for this wish, only a species list is presented.

### 2.4. Variation in the Structure of Knowledge between Genders

To analyze the variation of knowledge between genders in the structure of the system, the number of medicinal plants known by each informant was used. Regarding the variation of knowledge related to IUs, the plant-therapeutic target pair was used. The final analysis demonstrates not only how a person can treat diseases or how many plants they know but also how much knowledge they possess for each disease. In this way, a person can know* x *plant and* x* therapeutic targets but can know a much larger number of plants for each therapeutic target since some plants can be repeated in other treatments. For example, if the plant “Aroeira” was reported as a treatment for wounds and vaginal infections, there would be two pairs, “Aroeira-wound” and Aroeira-vaginal infection”, which would be considered IUs.

To verify whether women possess greater knowledge of medical plants, the numbers of medicinal plants cited between men and women were compared using the Wilcoxon-Mann-Whitney test for independent samples. The same test was used to compare the numbers of IUs between genders. This test was chosen because the data were not normally distributed according to the Shapiro-Wilk test.

### 2.5. Variation of the Functions of Knowledge between Genders

To analyze the variation of knowledge between genders in the functions of the system, the total therapeutic targets known to men and women were analyzed using the Wilcoxon-Mann-Whitney test. This test was chosen because the data do not have a normal distribution according to the Shapiro-Wilk test.

To test the function of the system, which encompasses that the redundancy of medicinal plants for each therapeutic target is greater among women than among men, only the targets that both genders knew were analyzed. Targets cited by only one gender were not included in the analysis due to the variation in the total number of therapeutic targets and species that each one knew.

Redundancy was not conventionally analyzed as proposed by Albuquerque and Oliveira [14] since the study was not focused on identifying the categories of redundancy. Redundancy was analyzed considering the total number of plants used to treat an identical therapeutic target. Thus, if women know ten plants for treating flu and men knew five for this therapeutic target, the redundancy would be higher among women for such a target; therefore, this analysis was performed for each target. Idiosyncratic targets were not considered to avoid errors in the data interpretation. For example, targets mentioned by one person were not considered unless the therapeutic target was quoted by a woman and/or two or more men, or vice versa. The utilitarian redundancy of the therapeutic targets was also analyzed, separating the targets affecting each gender: exclusive to women and exclusive to men.

The Wilcoxon-Mann-Whitney test was used for data analysis for independent samples since the data do not have a normal distribution.

### 2.6. Variation in the Transmission of Knowledge between Genders

To test the knowledge transmission between genders, several indicators of transmission were considered, such as the diversity of models when knowledge is obtained, the direction relative to the sex of the knowledge transmission and the sharing of knowledge in each gender.

To test if women learn from a wider range of models, the number of models that each informant indicated learning from was compared using the Wilcoxon-Mann-Whitney test. To verify whether the knowledge of women is transmitted more among women than among men, the number of models of the same gender and the number of models of both genders that obtained knowledge of medicinal plants were used. After nonnormality of the residuals was confirmed by the Kolmogorov-Smirnov test, the Kruskal-Wallis test was used, followed by a* posteriori* Dunn test.

To test if women share more information among themselves than men, the number of informants from each gender who share the same IU was analyzed, followed by the analysis of the therapeutic targets treated with medicinal plants that are shared between genders. Next, the therapeutic targets were divided according to their involvement with each gender: general (therapeutic targets that affect both genders), exclusive to men and exclusive to women. The four types of analysis were performed using an analysis of variance for two independent samples and the Wilcoxon-Mann-Whitney test after testing that the data were not normal using the Shapiro-Wilk test.

Analyses were performed in the software R version 3.2.4 revised [42], and all of the results that had values of p<0.05 were considered significant.

## 3. Results

A total of 232 medicinal plants were cited, of which 120 were identified to the species level and seven only to the genus level (see the appendix). There were 320 therapeutic targets recognized and 1833 IUs (plants and therapeutic targets) recorded Table 1.

The analyses revealed that there were significant differences in knowledge between genders Table 1, with men having greater knowledge of medicinal plants (W=23719, p<0.001), therapeutic targets (W=23651, p<0.001), and IUs (W=22940, p<0.01).

### 3.1. Utilitarian Redundancy of Medicinal Plants in Relation to Therapeutic Targets between Genders

Men have a greater knowledge of medicinal plants with utilitarian redundancy (W=8443, p<0.01) compared to therapeutic targets treated by both genders (median=5, mean=7.52, and SD=7.70), than women (median=3, mean=6.06, and SD=7.04).

There were no significant differences in the utilitarian redundancy of the therapeutic targets that only affect men (W=13.5, p>0.05) between men (median=1.5, mean=5, SD=8.44) and women (median=1, mean=1.83, and SD=2.71). There were also no significant differences in the utilitarian redundancy of therapeutic targets that affect only women (W=462.5, p>0.05) between men (median=1, mean=2.88, and SD=14.92) and women (median=2, mean=3.73, and SD=23.39).

### 3.2. Transmission of Knowledge of Medicinal Plants between Genders

There were no significant differences (W=17526, p>0.05) between the numbers of transmission models among men (median=2, mean=2.60, SD=1.90) and women (median=2, mean=2.84, SD=1.79). A total of 60 transmission models were recorded, such as “great-grandmother”, “great-grandfather”, “grandmother”, “grandfather”, “mother”, “father”, “uncle”, “aunt”, “brother”, “sister”, “niece”, “nephew”, “husband” and “wife”, and others, which have a relationship of kinship; and others who do not have a kinship relationship such as “mate”, “friend”, “street person”, “resident of the city”, “white” (referring to non-indigenous), “Recife”(reference to person living in the capital of the state of Pernambuco), “health professional”, “nurse”.

With respect to whether transmission models were directed by a specific gender, men learn more often with other men ($\overset{-}{x}$=1.127; SD=1.095) than with women ($\overset{-}{x}$=0.782, SD=0.872, Dunn: p<0.05) or with both genders ($\overset{-}{x}$=0.818, SD=0.676, Dunn: p<0.05) (H=53.098, p<0.05). Women learned more often from other women ($\overset{-}{x}$=1.836, SD=1.109) than with other men ($\overset{-}{x}$=0.945, SD=0.906, Dunn: p<0.05) or with people of both genders ($\overset{-}{x}$=0.563, SD=0.706, Dunn: p<0.05) (H=114.534, p<0.05).

There were significant differences (W=597220, p<0.05) between the numbers of IUs shared between men and women, where among the women; the same IU is better known among them (median=1, mean=2.53, and SD=5.69) than among the men (median=1, mean=2.28, and SD=5.12). With regard to the sharing of knowledge by dividing the therapeutic targets exclusive and non-exclusive to each gender, there were no significant differences in the shared knowledge of the targets affecting men (W=21, p>0.05), between men (median=2, mean=5, and SD=8.3) and women (median=1, mean=4.3, and SD=7.4). Similarly, there were no significant differences in the shared knowledge of the diseases that affect women (W=488, p>0.05) between men (median=1, mean=5.6, and SD=11.2) and women (median=2, mean=5, and SD=13.2). In the case of the therapeutic targets that are not exclusive to each gender, there was no significant difference between the numbers of men (median=1, mean=6.21, and SD=14.39) and women (median=1, mean=5.39, and SD=13.84) who have knowledge of each therapeutic target (W=43280, p>0.05).

## 4. Discussion

### 4.1. Structure of the Local Medical Systems Related to Gender

The women of indigenous community Fulni-ô had less knowledge of medicinal plants and IUs. As a consequence, the idea regarding the structure of the LMS related to gender was not supported. Does this mean that women within an LMS contribute less to the structure of the system? Women are important in the structure of LMSs since they possess a greater knowledge of medicinal plants [4, 27, 28], along with the men, according to the results of this and previous studies [5, 43, 44]. It has also been observed that knowledge of the structure of the system may be homogeneous between genders [45–47] and not unidirectional [6].

### 4.2. Function of the LMSs in relation to Gender

Women reported less knowledge of therapeutic targets treated with plants and had less utilitarian redundancy in their knowledge of medicinal plants compared to the knowledge of men, and there were no significant differences between genders in the knowledge of therapeutic targets exclusive to each. Therefore, our hypothesis related to the function of the LMSs is not supported. According to the first utilitarian redundancy models related to system functionality, the greater the utilitarian redundancy in a system is, the greater the resilience of the system, since the loss of a species would not greatly damage the system, as others could replace it [5, 12, 48]. This study shows that regarding the redundant knowledge of species to treat certain therapeutic targets, the men within the LMSs have a greater contribution to the resilience of the system. The opposite was observed in the knowledge of women since systems that have low redundancy can generate a gap in the treatment of specific therapeutic targets when faced with a loss. However, the contribution of redundancy by gender may vary by community. For example, in the case of the community of Tsimani in Bolivia, the redundancy between genders was similar, with the exception of some therapeutic targets [49]. Our results indicate that when functions are analyzed with respect to the type of condition (exclusive to men or women), there is no variation in redundancy. Thus, gender variation factors act not only at a structural level but also at a functional level in the system.

### 4.3. The Transmission of Knowledge Related to Gender as an Important Factor for the Resilience of LMSs

The results do not support the hypothesis that women obtain knowledge with more models of transmission than men. This hypothesis proposed that since women are responsible for the care of the family, they would consult a greater diversity of models compared to men because they would be exposed to a greater number of disease events compared to men in their search for treating these diseases. Considering that disease events of individuals or other people, either experienced or shared, are one of the most important stimuli and/or contexts of medicinal plant knowledge [20] and that the knowledge to treat them is acquired within the family first, as a child, with other sources being used as the individual reaches adulthood [50], our results show that those stimuli may not differ between genders, which is explained by the fact that both have an interest in the pursuit of knowledge of different models to treat diseases, taking into account that our informants were married men and women, which is why the stimulus in the presence of the disease may possibly be shared.

In our study, women learn more from other women than with men, and men learn more from other men than from women, which supports our hypothesis regarding a knowledge transmission related to gender. Our results show the selection of the learning model relative to gender based on the future social role that the apprentice will have in society; therefore, men learn from men and women from women [29]. In a study on medical plant knowledge, Henrich and Broesh [29] did not find model selection related to sex since the members of the community learning about medicinal plants resorted frequently to women regardless of the gender of the apprentice. Taking into account that our interviewees are over the age of 18 and that studies related to model selection in relation to sex are actually more related to the stage of childhood [19, 51], we suggest that the selection of the model remains until adulthood because of the social role related to gender and that the knowledge that makes up the system is being incorporated at an early age relative to gender.

The results obtained regarding knowledge sharing support our hypothesis that women share more knowledge among themselves than among men. Regarding the proposal that the sharing of knowledge is an important factor in the resilience of the system [12] women are important in maintaining the function of the system since they share more knowledge among themselves and thus socialize knowledge more than men. From the perspective of sharing knowledge over time, one may assume that the lack of socialization among men has led them to experience and possess greater knowledge, as observed in the structure and function results. In contrast, the knowledge of women is maintained as structure in the system, being diversified by the socialization of knowledge between them, which can contribute to their individual autonomy to manage health problems.

From a theoretical point of view, we assume that psychological characteristics of the gender may be directing the differences in socialization of knowledge between them. In this regard, Bunce and Peterson [52] indicate that there are differences in the socialization of gender when faced with negative events and suggest that women show sociability and a pessimistic reaction that makes them be more concerned regarding their health and wellbeing, while men react with shyness and discomfort in the face of a social situation.

### 4.4. Final Considerations

The indigenous community Fulni-ô is hermetic. It does not share information related to their social activities within their sacred ritual with nonindigenous people. The ritual is what governs their culture, so there is no evidence of cultural norms that make a difference in the knowledge of medicinal plants among men and women in the village, as has been suggested in some communities in Ethiopia where knowledge of Medicinal plants is directed only to men [43]. Ethnological studies of the Fulni-ô have suggested that men are taught the secrets of rituals, knowledge that cannot be revealed to women. At birth the men are given a tribal name referring to a plant and are the only ones that can approach the sacred tree “Juazeiro” [53], which shows the importance of men in the culture. Given the above, our results may be influenced by the importance of men in the culture having a greater knowledge of medicinal plants. However, the observed results show that despite possible differences within the culture, both genders have a participation in the local medical system. Therefore, it is suggested that, in order to understand the gender-local medical system relationship, it is important to know the social norms, to analyze the structure and functionality of knowledge in relation to each gender.

## 5. Conclusions

**Figure 1:**
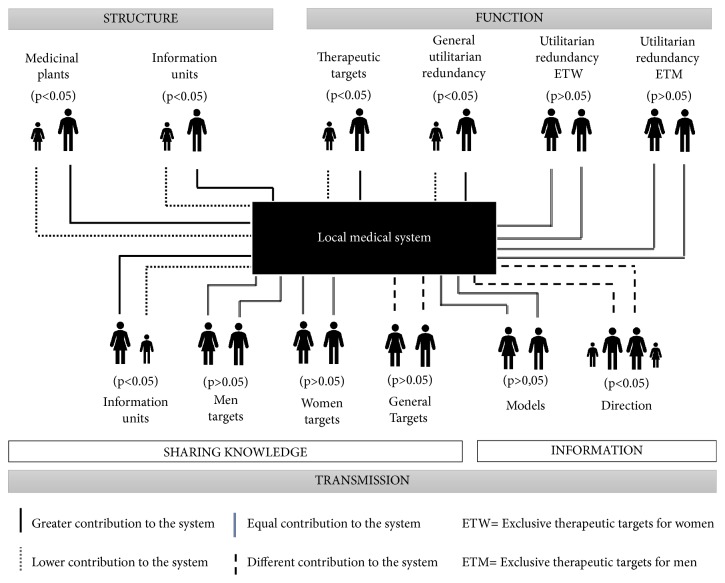
Influence of gender on the resilience of the local medical system Fulni-ô: structure and functionality.

Considering our results in general (Figure 1), we suggest that, in the LMSs, men can a greater contribution to the resilience of a system because they provide a greater knowledge of the structure and function of the Fulni-ô medical system. However, from a systemic point of view, women have an important contribution to the same functionality since they socialize more knowledge than men and therefore contribute more to maintaining the functions. However, both genders contribute to the functionality of the system and to the flexibility it needs for its resilience to disturbance. To analyze the differences between genders within the local medical systems, it is important to observe the results from a systemic point of view (structure, function and functionality).

Further, our results show the importance of not breaking down gender in studying resilience of social-ecological systems since this approach permits an understanding of different relationships of the resource with social actors, allowing a greater understanding of their position in decision making. The importance of gender in social-ecological systems has also been indicated in the recognition of ecosystem services, which has demonstrated that differences exist between genders [54, 55].

Given the importance of not breaking down gender in studying the resilience of systems, the study of governance in social-ecological systems, which includes all aspects of rules and regulations that determine when and how people operate in the system, along with the different types of institutions that influence or determine how people behave [8, 56], should integrate the genders of the social actors.

## Figures and Tables

**Table 1:** Structure and function of the local medical system of the indigenous community Fulni-ô (northeastern Brazil) in relation to gender.

	Total		Men			Women			Analysis	
	Men	Women	Median	Mean	SD	Median	Mean	SD	W	p

Medicinal plants	196	168	9	10.35	6.09	7	8.44	5.47	23719	0.001∗
therapeutic targets	265	216	10	10.91	6.29	7	9.08	6.17	23651	0.001∗
Information units	1261	981	13	14.61	9.74	9	12.58	9.90	22940	0.01∗

∗p<0.05 significant.

**Table 2:** List of species.

Local name	Specie or gender and familiy	Vouchers
Abacate	*Persea americana* Mill. (Lauraceae)	Comercial
Abacaxi	*Ananas comosus* (L.) Merr. (Bromeliaceae)	Comercial
Abóbora	*Cucurbita sp*. (Cucurbitaceae)	Comercial
Azeitona preta	*Syzygium cumini* (L.) Skeels (Myrtaceae)	218 Torres, W.
Acerola	*Malpighia emarginata* DC. (Malpighiaceae)	233 Torres, W.
Alastrado	*Pilosocereus gounellei* (F.A.C. Weber) Byles & Rowley (Cactaceae)	197 Torres, W.
Alecrim	*Lippia gracilis* Schauer (Verbenaceae)	Santos, A. 90287 registro
Alecrim (cultivado)	*Rosmarinus officinalis* L. (Lamiaceae)	193 Torres, W.
Alenta cavalo	*Pfaffia glomerata* (Spreng.) Pedersen (Amaranthaceae)	174 Torres, W.
Alfavaca	*Ocimum campechianum* Mill. (Lamiaceae)	175 Torres, W.
Alfavaca de vaqueiro	*Ocimum americanum* L. (Lamiaceae)	227 Torres, W.
Alface	*Lactuca sativa* L. (Asteraceae)	Comercial
Algaroba	*Prosopis juliflora* (Sw.) DC. (Fabaceae)	219 Torres, W.
Algodão	*Gossypium hirsutum* L. (Malvaceae)	173 Torres, W.
Alho	*Allium sativum* L. (Amaryllidaceae)	Comercial
Alpiste	*Phalaris canariensis* L. (Poaceae)	Comercial
Ameixa	*Prunus sp.*(Rosaceae)	Comercial
Anador	*Emilia fosbergii* Nicolson (Asteraceae)	231 Torres, W.
Andú	*Cajanus cajan* (L.) Huth (Fabaceae)	237 Torres, W.
Angico de caroço	*Anadenanthera colubrina* var. *cebil*(Griseb.) Altschul (Fabaceae)	209 Torres, W.
Angico manjolo	*Anadenanthera colubrina* var. *cebil*(Griseb.) Altschul (Fabaceae)	203 Torres, W.
Aniz estrelado	*Illicium verum* Hook. f. (Schisandraceae)	Comercial
Arapiraca	*Senegalia sp.*(Fabaceae)	207 Torres, W.
Arió	*Cissus simsiana* Schult. & Schult.f. (Vittaceae)	200 Torres, W.
Arnica	*Arnica sp.*(Asteraceae)	Comercial
Aroeira	*Myracrodruon urundeuva* Allemão (Anacardiaceae)	Santos, A. 90266 registro
Arruda	*Ruta graveolens* L. (Rutaceae)	Comercial
Aveloi	*Euphorbia tirucalli* L. (Euphorbiaceae)	201 Torres, W.
Babão (Mandacaru)	*Cereus jamacaru* DC. (Cactaceae)	217 Torres, W.
Babosa	*Aloe vera* (L.) Burm.f. (Asphodelaceae)	191 Torres, W.
Bananeira	*Musa sp.*(Musaceae)	Comercial
Baraúna	*Schinopsis brasiliensis* Engl. (Anacardiaceae)	168 Torres, W.
Barriguda	*Ceiba glaziovii* (Kuntze) K. Schum. (Malvaceae)	212 Torres, W.
Berinjela	*Solanum melongena* L. (Solaneaceae)	Comercial
Beterraba	*Beta vulgaris* L. (Amaranthaceae)	Comercial
Bom nome	*Maytenus rigida *Mart. (Celastraceae)	Santos, A. 90273 registro
Brócolis	*Brassica oleracea* var. *italica* Plenck (Brassiacaceae)	Comercial
Café	*Coffea arabica* L. (Rubiaceae)	Comercial
Cajueiro branco	*Anacardium occidentale* L. (Anacardiaceae)	190 Torres, W.
Cajueiro roxo	*Anacardium occidentale* L. (Anacardiaceae)	206 Torres, W.
Calacancão	*Argemone mexicana* L. (Papaveraceae)	157 Torres, W.
Camomilla	*Matricaria chamomilla* L. (Asteraceae)	Comercial
Canela	*Cinnamomum verum* J. Presl (Lauraceae)	Comercial
Canela de nambu	*Ruellia asperula* (Mart. ex Ness) Lindau (Acanthaceae)	Santos, A. 90272 registro
Canha	*Saccharum officinarum* L. (Poaceae)	Comercial
Canha de macaco	*Maranta divaricata* Roscoe (Maranthaceae)	163 Torres, W.
Capim santo	*Cymbopogon citratus* (DC.) Stapf (Poaceae)	180 Torres, W.
Catingueira	*Caesalpinia pyramidalis* Tul. (Fabaceae)	Santos, A. 90284 registro
Cebola branca	*Allium cepa* L. (Amaryllidaceae)	Comercial
Cenoura	*Daucus carota* L. (Apiaceae)	Comercial
Chia	*Salvia hispanica* L. (Lamiaceae)	Comercial
Chuchu	*Sechium edule* (Jacq.) Sw. (Cucurbitaceae)	Comercial
Ciriguela	*Spondias purpurea* L. (Anacardiaceae)	182 Torres, W.
Coco	*Cocos nucifera* L. (Arecaceae)	Comercial
Coroa de frade	*Melocactus zehntneri* (Britton & Rose) Luetzelb. (Cactaceae)	216 Torres, W.
Couve flor	*Brassica oleracea* var. *botrytis *L. (Brassicaceae)	Comercial
Craibeira	*Tabebuia aurea* (Silva Manso) Benth. & Hook.f. ex S. Moore (Bignoniaceae)	213 Torres, W.
Caruá	*Neoglaziovia variegata* (Arruda) Mez (Bromeliaceae)	224 Torres, W.
Crista de galo	*Heliotropium elongatum* (Lehm.) I. M. Johnst. (Boraginaceae)	236 Torres, W.
Endro	*Anethum graveolens* L. (Apiaceae)	Comercial
Erva cidreira	*Lippia alba* (Mill.) N.E.Br. ex P. Wilson (Verbenaceae)	181 Torres, W.
Erva doce	*Anisum officinale* DC. (Apiaceae)	Comercial
Espinheiro branco	*Senegalia bahiensis* (Benth.) Seigler & Ebinger (Fabaceae)	185 Torres, W.
Espinheiro vermelho	*Senegalia riparia* (Kunth) Britton & Rose ex Britton & Killip (Fabaceae)	205 Torres, W.
Fedegosso	*Senna occidentalis* (L.) Link. (Fabaceae)	196 Torres, W.
Feijão bravo	*Cynophalla hastata* (Jacq.) J. Presl (Capparaceae)	222 Torres, W.
Fumo	*Nicotiana tabacum* L. (Solanaceae)	Comercial
Carrapicho de agulha	*Bidens pilosa* L. (Asteraceae)	159 Torres, W.
Gengibre	*Zingiber officinale* Roscoe (Zingeberaceae)	Comercial
Genipapo	*Genipa americana* L. (Rubiaceae)	225 Torres, W.
Girassol	*Helianthus annuus* L. (Asteraceae)	Comercial
Goiabeira	*Psidium guajava* L. (Myrtaceae)	188 Torres, W.
Hortelão da folha grande	*Plectranthus amboinicus* (Lour.) Spreng. (Lamiaceae)	211 Torres, W.
Imburana de cambão	*Commiphora leptophloeos* (Mart.) J.B. Gillett. (Burseraceae)	167 Torres, W.
Juazeiro	*Ziziphus joazeiro* Mart. (Rhamnaceae)	Santos, A. 90269 registro
Junça	*Libidibia ferrea* (Mart. ex Tul.) L.P. Queiroz (Fabaceae)	204 Torres, W.
Junco 3	*Eleocharis sp.*(Cyperaceae)	164 Torres, W.
Jurema branca	*Mimosa tenuiflora* (Willd.) Poir. (Fabaceae)	Santos, A. 90286 registro
Jurema de caboclo	*Vitex agnus-castus* L. (Verbenaceae)	166 Torres, W.
Jurema preta	*Mimosa tenuiflora* (Willd.) Poir. (Fabaceae)	Santos, A. 90285 registro
Leucena	*Leucaena leucocephala* (Lam) de Wit (Fabaceae)	177 Torres, W.
Limão	*Citrus limon* (L.) Osbeck (Rutaceae)	Comercial
Louro	*Ocimum gratissimum* L. (Lamiaceae)	170 Torres, W.
Macambira	*Bromelia laciniosa* Mart. ex Schult. & Schult.f. (Bromeliaceae)	198 Torres, W.
Mamão	*Carica papaya* L. (Caricaceae)	194 Torres, W.
Mandioca	*Manihot esculenta* Crantz (Euphorbiaceae)	Comercial
Manguera	*Mangifera indica* L. (Anacardiaceae)	208 Torres, W.
Manjericão	*Ocimum campechianum* Mill. (Passifloraceae)	165 Torres, W.
Maracujá	*Passiflora edulis* Sims (Passifloraceae)	176 Torres, W.
Maracujá de estralo	*Passiflora foetida* L. (Passifloraceae)	228 Torres, W
Mastruz	*Dysphania ambrosioides* (L.) Mosyakin & Clemants (Amaranthaceae)	169 Torres, W.
Melão	*Cucumis melo* L. (Cucurbitaceae)	Comercial
Melão de São caetano	*Momordica charantia* L. (Cucurbitaceae)	187 Torres, W.
Meloncia	*Citrullus lanatus* (Thunb.) Matsum. & Nakai (Cucurbitaceae)	Comercial
Milho	*Zea mays* L. (Poaceae)	179 Torres, W.
Mororó	*Bauhinia sp.*(Fabaceae)	Santos, A. 90276 registro
Mucuna	*Dioclea grandiflora* Mart. ex Benth. (Fabaceae)	239 Torres, W.
Mulungu	*Erythrina velutina* Willd. (Fabaceae)	223 Torres, W.
Mussambê	*Tarenaya spinosa* (Jacq.) Raf. (Capparaceae)	230 Torres, W.
Nabo	*Brassica rapa* L. (Brassicaceae)	Comercial
Ouricuri	*Syagrus coronata* (Mart.) Becc. (Arecaceae)	214 Torres, W.
Papaconha	*Sida cordifolia* L. (Malvaceae)	242 Torres, W.
Pau brasil	*Caesalpinia echinata* Lam. (Fabaceae)	229 Torres, W.
Pau darco	*Cordia trichotoma* (Vell.) Arráb. ex Steud. (Boraginaceae)	210 Torres, W.
Pau darco roxo	*Handroanthus impetiginosus*(Mart. ex DC.) Mattos (Bignoniaceae)	221 Torres, W.
Pau ferro	*Chloroleucon foliolosum* (Benth.) C.P. Lews (Fabaceae)	Santos, A. 90278 registro
Pega pinto	*Boerhavia diffusa* L. (Nyctagininaceae)	161 Torres, W.
Pepino	*Cucumis sativus* L. (Cucurbitaceae)	Comercial
Pereiro	*Aspidosperma pyrifolium* Mart. (Apocynaceae)	Santos, A. 90274 registro
Pinha	*Annona muricata* L. (Annonaceae)	172 Torres, W.
Pinhão branco	*Jatropha mollissima* (Pohl) Baill. (Euphorbiaceae)	186 Torres, W.
Pinhão bravo	*Jatropha mollissima* (Pohl) Baill. (Euphorbiaceae)	Santos, A. 90263 registro
Pinhão rastreiro	*Jatropha ribifolia* (Pohl) Baill. (Euphorbiaceae)	226 Torres, W.
Pinhão roxo	*Jatropha gossypiifolia* L. (Euphorbiaceae)	Santos, A. 90264 registro
Quebra pedra	*Phyllanthus amarus* Schumach. & Thonn. (Euphorbiaceae)	195 Torres, W.
Quipá (folha de palma)	*Tacinga inamoena* (K. Schum.) N.P. Taylor & Stuppy (Cactaceae)	215 Torres, W.
Quixaba	*Sideroxylon obtusifolium*(Roem. & Schult.) T.D. Penn. (Sapotaceae)	Santos, A. 90268 registro
Rabo de raposa	*Harrisia adscendens* (Gürke) Britton & Rose (Cactaceae)	192 Torres, W.
Romã	*Punica granatum* L. (Lythraceae)	234 Torres, W.
Sabogueira	*Solanum stipulaceum* Willd. ex Roem. & Schult. (Solanaceae)	241 Torres, W.
Sambacaitá	*Hyptis pectinata* (L.) Poit. (Lamiaceae)	158 Torres, W.
Trapiá	*Crateva tapia* L. (Capparaceae)	171 Torres, W.
Umbu	*Spondias tuberosa* Arruda (Anacardiaceae)	Santos, A. 90267 registro
Urtiga	*Cnidoscolus urens* (L.) Arthur (Euphorbiaceae)	162 Torres, W.
Vassourinha de botão	*Borreria verticillata* (L.) G. Mey. (Rubiaceae)	232 Torres, W.
Velame	*Croton heliotropiifolius* Kunth (Euphorbiaceae)	Santos, A. 90265 registro
Velandinho	*Croton tetradenius* Baill. (Euphorbiaceae)	238 Torres, W.

## Data Availability

The data used to support the findings of this study are included within the article and can be solicited by request to the authors.

## Appendix

See Table 2.
